# Molecular Simulation Study of Water–Rock Interfaces During Supercritical CO_2_ Sequestration

**DOI:** 10.3390/molecules31020268

**Published:** 2026-01-13

**Authors:** Yuanzi Yan, Yunfeng Fan, Peng Zhang

**Affiliations:** 1 Shaanxi Key Laboratory of Higher Education Institutions for Intelligent Prevention and Control of Coal Mine Disasters, Shaanxi Energy Institute, Xianyang 712000, China; 2 Geology Research Institute, China National Logging Corporation, Xi’an 710077, China; zycjfanyf@cnpc.com.cn; 3 CCTEG Xi’an Research Institute (Group) Co., Ltd., Xi’an 710077, China; zhangpeng2@cctegxian.com

**Keywords:** supercritical CO_2_ sequestration, mineral wettability, interfacial electrostatic interactions, confined water structure, molecular dynamics simulation

## Abstract

Understanding how supercritical CO_2_ and water interact with mineral surfaces is essential for predicting the stability and sealing performance of geological storage formations. Yet, the combined effects of mineral surface chemistry and confined pore geometry on interfacial structure and fluid dynamics remain insufficiently resolved at the molecular scale. In this study, molecular dynamics simulations were employed to quantify how methylated SiO_2_, hydroxylated SiO_2_, and kaolinite regulate CO_2_–H_2_O interfacial behavior through variations in wettability and electrostatic interactions. The results show a clear hierarchy in water affinity across the three minerals. On methylated SiO_2_, the water cluster remains spherical and poorly anchored, with a contact angle of ~140°, consistent with the weakest water–surface Coulomb attractions (only −400 to −1400 kJ/mol). Hydroxylated SiO_2_ significantly enhances hydration, forming a cylindrical water layer with a reduced contact angle of ~61.3° and strong surface–water electrostatic binding (~−18,000 to −20,000 kJ/mol). Kaolinite exhibits the highest hydrophilicity, where water forms a continuous bridge between the two walls and the contact angle further decreases to ~24.5°, supported by the strongest mineral–water electrostatic interactions (−23,000 to −25,000 kJ/mol). Meanwhile, CO_2_–water attractions remain moderate (typically −2800 to −3500 kJ/mol) but are sufficient to influence CO_2_ distribution within the confined domain. These findings collectively reveal that surface functionalization and mineral type govern interfacial morphology, fluid confinement, and electrostatic stabilization in the sequence methylated SiO_2_ < hydroxylated SiO_2_ < kaolinite. This molecular-level understanding provides mechanistic insight into how mineral wettability controls CO_2_ trapping, fluid segregation, and pore-scale sealing behavior in subsurface carbon-storage environments.

## 1. Introduction

Carbon dioxide emissions continue to rise alongside global energy consumption, intensifying concerns over climate change and atmospheric carbon accumulation. While renewable technologies are expanding, fossil fuels remain deeply embedded in the global energy structure, meaning large-scale carbon mitigation strategies are urgently required. Among the available approaches, geologic carbon sequestration has emerged as one of the most practical and scalable solutions, capable of permanently storing CO_2_ in subsurface environments [1,2,3]. In particular, the injection of CO_2_ under supercritical conditions into deep saline aquifers, depleted hydrocarbon reservoirs, and basaltic formations has attracted growing research interest due to the high storage capacity, favorable physicochemical properties of supercritical CO_2_ (scCO_2_), and the potential for long-term mineral trapping. As international carbon neutrality goals accelerate deployment of such technologies, understanding the behavior of scCO_2_ within complex geologic settings is becoming increasingly important [4,5].

Recent advances have significantly expanded understanding of how supercritical CO_2_ interacts with subsurface minerals, pore networks, and confined aqueous phases, and current research may be broadly grouped into experimental characterization, shale- and sandstone-specific investigations, reactive transport modeling, and long-term evaluation studies. In the first category, many laboratory studies have focused on observing mineral dissolution, secondary precipitation and pore structure evolution under reservoir-like temperature and pressure conditions [6,7,8,9,10]. Ma and coauthors found that scCO_2_ exposure in coal and organic-rich systems can substantially increase pore volume and specific surface area in particular size ranges, indicating creation or enlargement of micro- and mesopores that could alter transport pathways [11]. Medina and colleagues combined gas adsorption and NMR data on Wolfcamp shale samples to show that short-term scCO_2_ injection redistributes pore size populations from subnanometer to micron scales and modifies mass transport rates in a lithology-dependent manner [9]. Bai’s experimental work demonstrated that scCO_2_ immersion not only modifies pore geometry in layered shales but also measurably weakens mechanical strength, implying coupled hydro-chemical–mechanical effects that may accelerate microfracture development and enhance connectivity [8]. Broader experimental syntheses and targeted studies reported by Lyu and others document systematic patterns of adsorption-driven swelling or shrinkage in clay-rich intervals and highlight that mineralogical composition controls whether scCO_2_ contact leads to net pore opening or pore clogging [12]. Recent laboratory extractions and imaging studies further reveal that the direction and magnitude of porosity and permeability change depend strongly on initial mineralogy, brine salinity, exposure duration and the presence of organic matter, leaving a complex, sample-specific fingerprint that must be accounted for when scaling observations to reservoir behavior [13,14,15,16,17].

Computational studies have increasingly complemented laboratory work by revealing mechanistic details and scaling pathways that are difficult to capture experimentally. Reactive-transport simulators such as CrunchFlow and PHREEQC have been used to reproduce coupled CO_2_ dissolution, pH evolution, ion exchange and mineral dissolution/precipitation over field-relevant timescales, demonstrating how geochemical fronts migrate from injection points and drive localized porosity changes [18,19,20,21,22]. At the pore scale, new numerical frameworks that explicitly resolve multiphase flow and chemistry inside realistic pore geometries have started to quantify how wetting changes and mineral reactions reorganize flow paths; for example, a recently developed pore-scale solver couples advective–diffusive transport with surface reaction kinetics to link mineral loss or gain with permeability evolution. Phase-field and lattice-based models have also been adapted to simulate precipitation and dissolution in heterogeneous packings and basalts, showing that microscale heterogeneities can generate non-intuitive sealing or channelization patterns even when bulk chemistry predicts the opposite. At smaller length scales, atomistic simulations including molecular dynamics and density functional theory have provided detailed pictures of CO_2_ adsorption, competitive water adsorption, and ion coordination at mineral and clay surfaces, and these results have been used to parameterize sorption isotherms and reaction-rate laws for continuum models [23,24,25,26,27]. Taken together, the modeling literature highlights two important points for scCO_2_ storage: first, process coupling across scales is essential because adsorption and surface chemistry at the angstrom-to-nanometer scale influence pore-scale transport, which, in turn, controls field-scale trapping; second, uncertainty in kinetic parameters, transport upscaling, and the treatment of organic-rich phases remain major impediments to reliable predictive simulations.

Molecular-scale simulations have become an increasingly powerful tool because they capture interfacial behavior and confinement effects that cannot be isolated experimentally or resolved in continuum models. These methods make it possible to directly observe competitive adsorption, hydration structure rearrangement, and wetting transitions under reservoir-relevant pressure and temperature [28,29,30]. Compared with laboratory tests, molecular dynamics offers full control over mineral chemistry and pore geometry, allowing cause-and-effect relationships to be quantified rather than inferred. Such atomistic insight has proven essential for refining thermodynamic descriptions, improving surface reaction models, and constraining parameters used in pore-scale and reactive-transport simulations. However, uncertainties persist regarding how mineral wettability and sediment type regulate the coexistence and mobility of supercritical CO_2_ and water in confined pores. To address this knowledge gap, the present study employs molecular dynamics to construct hydroxylated and methylated silica slit pores alongside kaolinite interfaces. Through comparative simulations, we examine how these distinct mineral surfaces influence interfacial structure, adsorption behavior, and phase distribution of scCO_2_ and water.

## 2. Simulation Models and Methods

**Figure 1:**
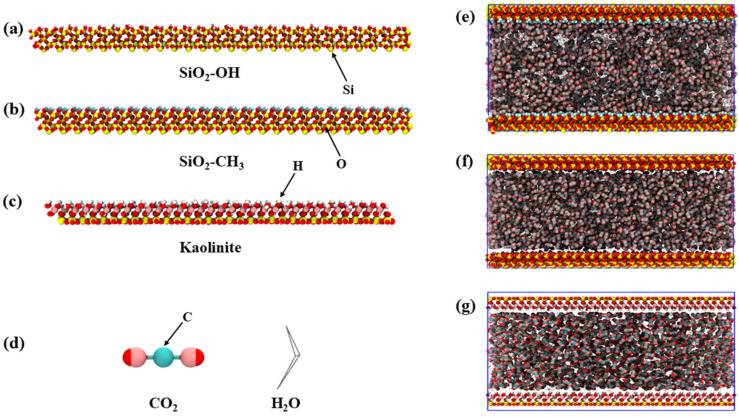
Structural models of (**a**) hydroxylated/(**b**) methylated silica and (**c**) kaolinite. (**d**) Five-site CO_2_ and water molecular models. Initial configurations of mixed CO_2_–water systems confined within (**e**) methylated silica, (**f**) hydroxylated silica, and (**g**) kaolinite slit pores.

To examine how sediment type regulates interfacial behavior between supercritical CO_2_ and water, three mineral substrates were constructed to represent typical reservoir lithologies. Silica (SiO_2_) was selected as the quartz endmember, while kaolinite was chosen to reflect clay-rich environments. To further capture the influence of surface chemistry, the silica model was built in two forms: a hydrophilic configuration where surface dangling bonds were fully terminated with hydroxyl groups, and a hydrophobic counterpart generated by replacing these groups with methyl terminations (Figure 1a,b). The kaolinite slab inherently exhibits hydrophilic character and was therefore kept chemically untreated (Figure 1c). The three mineral substrates were selected to represent typical endmembers of surface chemistry and wettability in geological CO_2_ storage systems. Hydroxylated SiO_2_ corresponds to quartz-rich sandstone reservoirs, where surface silanol groups dominate under water-saturated conditions. Methylated SiO_2_ was introduced as a hydrophobic reference surface, mimicking silica surfaces modified by organic coatings or hydrocarbon exposure, which have been reported in reservoir and caprock environments. Kaolinite represents clay-rich lithologies such as shales and mudstones, which are widely distributed in caprocks and exhibit strong hydrophilicity due to structural hydroxyl groups and layer charge. Together, these substrates provide a simplified yet physically meaningful framework to systematically evaluate how mineral surface chemistry regulates CO_2_–H_2_O interfacial structure under confinement. Supercritical CO_2_ molecules were modeled using a rigid five-site representation, while water was described using a four-site molecular model that has been widely applied under high-pressure and confined conditions (Figure 1d). Each mineral substrate was used to construct a slit-pore system with initial box dimensions of approximately 14.5 nm × 5.0 nm × 8.35 nm, corresponding to an effective pore width of about 7 nm. Within each pore configuration, CO_2_ and water were introduced as a mixed phase with a number ratio close to 1:2, reflecting compositions relevant to subsurface sequestration environments (Figure 1e–g). It should be noted that the CO_2_–H_2_O number ratio of approximately 1:2 adopted in this work does not aim to represent a unique reservoir-scale saturation state, such as a fully water-saturated saline aquifer or a depleted hydrocarbon field with residual fluids. Instead, this intermediate mixed-phase configuration was selected to ensure the coexistence of CO_2_ and water within the confined pore, enabling direct comparison of mineral-dependent interfacial behavior under identical thermodynamic conditions. At the pore scale, local fluid compositions can differ markedly from bulk averages due to wettability contrasts, capillary effects, and confinement. The present simulations therefore emphasize mechanistic insights into wettability control, electrostatic stabilization, and interfacial morphology, while acknowledging that absolute phase proportions may vary across different geological CO_2_ storage scenarios.

Si atoms are shown in yellow, O atoms in red, H atoms in white, and C atoms in green, while water molecules are depicted as gray line representations for visual clarity. For the kaolinite system, a slightly smaller substrate size was adopted due to the necessity of edge cutting and edge termination. The CO_2_/H_2_O number ratio and all molecular models were kept consistent with the silica systems to ensure comparability of interfacial behaviors. In realistic subsurface sequestration environments, formation water typically contains various dissolved ions, which can influence water structure, interfacial electrostatics, and CO_2_–H_2_O interactions. In the present study, ionic species were not explicitly included in order to isolate the intrinsic effects of mineral surface chemistry and wettability on confined CO_2_–water interfacial behavior. This simplified model allows a clearer interpretation of how surface functionalization and mineral type govern fluid morphology and electrostatic stabilization at the molecular scale. While the absence of ions may affect the quantitative strength of interfacial interactions, the qualitative trends in wettability, fluid segregation, and interfacial structure are expected to remain robust. The potential influence of salinity and specific ion effects will be addressed in future work to extend the applicability of the model to more complex reservoir conditions.

Silica surfaces were parameterized using the CHARMM27 force field [31], whereas kaolinite was represented with the CLAYFF force field, which is widely applied for layered aluminosilicate systems [32]. Water molecules were modeled with TIP4P-2005; this four-site representation employs virtual interaction points and was held rigid through the SETTLE constraint scheme [33]. Supercritical CO_2_ was described using the EPM2 model [34,35]. Interatomic Lennard–Jones interactions followed the Lorentz–Berthelot mixing convention. A cutoff distance of 1.20 nm was applied to short-range van der Waals and Coulomb terms, while long-range electrostatics were treated using the particle–mesh Ewald (PME) method. All simulations were carried out with periodic boundary conditions imposed in the x, y and z directions, and an integration time step of 2 fs. Prior to production runs, the systems underwent steepest-descent energy minimization, followed by an isothermal–isobaric equilibration stage of 500 ps at 323.15 K and 150 bar. During this phase, temperature and pressure were regulated by the V-rescale thermostat and Berendsen barostat, using relaxation constants of 0.1 ps and 2 ps, respectively, with semi-isotropic pressure control. After equilibration, production simulations were performed in the NPT ensemble for 15 ns at the same thermodynamic conditions. The Nosé–Hoover thermostat and Parrinello–Rahman barostat were employed during this stage, with coupling times of 2 ps and 4 ps, respectively, while maintaining semi-isotropic pressure treatment. All molecular dynamics calculations were performed using the GROMACS v5.0.7 simulation package.

## 3. Results and Discussion

**Figure 2:**
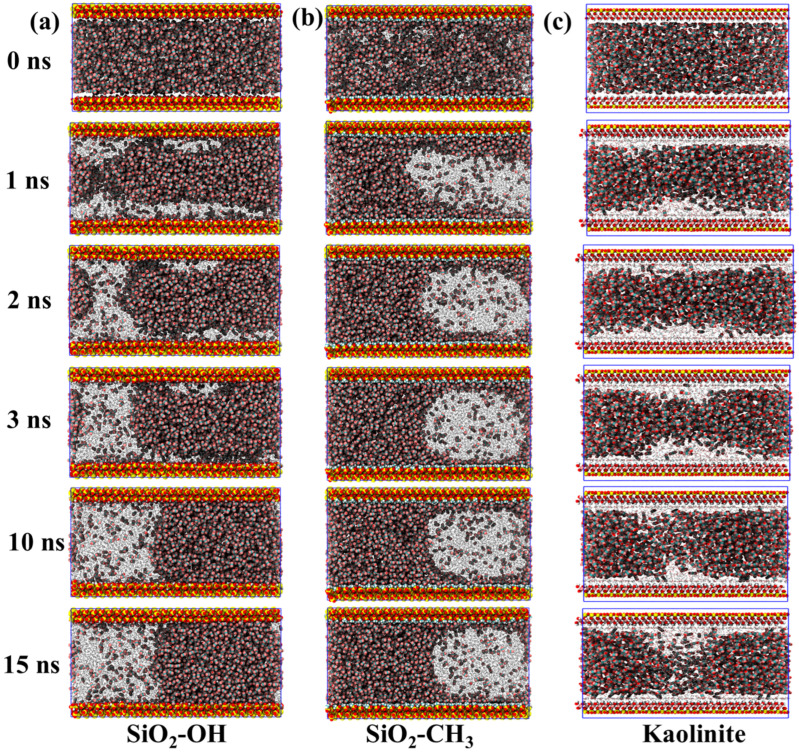
Structural evolution of CO_2_–water interfaces within (**a**) hydroxylated, (**b**) methylated SiO_2_ and (**c**) kaolinite systems.

Figure 2 presents the temporal evolution of interfacial configurations formed between supercritical CO_2_ and water within three representative sedimentary pore environments, namely hydroxylated silica, methylated silica, and kaolinite. For the hydroxylated SiO_2_ system (Figure 2a), the interface evolution between CO_2_ and water exhibits a clear wettability-controlled restructuring process. At the beginning of the simulation (0 ns), water and CO_2_ are initially dispersed throughout the slit pore without obvious phase separation. As time progresses, the spatial distribution of CO_2_ becomes increasingly confined toward the pore center, while water begins to migrate toward the mineral walls due to strong surface hydrophilicity and hydrogen-bond interactions. By approximately 2 ns, a distinct adsorbed water layer has formed along both SiO_2_ surfaces, and a continuous water bridge emerges, connecting the upper and lower boundaries of the pore space. With further simulation time, this water bridge thickens and expands laterally, progressively compressing the CO_2_ phase. Water molecules originally trapped at the CO_2_–solid interface are displaced outward, allowing CO_2_ molecules to come into direct contact with the silica surface. By around 10 ns, the system reaches a stable configuration characterized by pronounced phase separation: a water-dominated region occupies one side of the pore, while CO_2_ occupies the opposite side. A small population of dissolved CO_2_ remains detectable within the aqueous phase, reflecting its finite solubility under supercritical conditions.

In contrast, the interfacial evolution in the methylated SiO_2_ system displays the opposite wetting behavior compared with the hydroxylated surface (Figure 2b). Owing to the hydrophobic character of the functionalized silica, water does not spread along the pore walls but is progressively displaced by CO_2_. As early as 1 ns, most of the pore volume is already occupied by CO_2_, while water is compressed into a localized cluster near the central region of the slit pore. At the same time, a stable CO_2_ adsorption layer develops along the SiO_2_ surface, highlighting the strong affinity between the nonpolar substrate and the supercritical fluid. As the simulation continues, the water domain becomes further confined and gradually reshapes into an irregular, nearly spherical droplet fully surrounded by CO_2_, without forming direct contact with the solid surface. Throughout the subsequent trajectory (approximately 2–15 ns), both the morphology and spatial position of the droplet remain relatively unchanged, suggesting that the system reaches a metastable wetting configuration. A small number of CO_2_ molecules remain dissolved within the water droplet, consistent with the partial miscibility of supercritical CO_2_ under reservoir-like thermodynamic conditions. The final water droplet diameter is slightly smaller than the pore width, further confirming the stable encapsulated configuration.

Compared with silica, the kaolinite system exhibits a distinctly different interfacial evolution despite its similar hydrophilic properties (Figure 2c). At the beginning of the simulation, the entire mineral surface becomes rapidly coated with a continuous layer of water molecules, rather than the partial and gradual adsorption observed on hydroxylated SiO_2_. CO_2_ is initially confined to the central region of the slit pore, forming a flat, block-like phase that remains separated from the solid surface. As the system evolves, the central CO_2_ zone undergoes progressive structural disruption driven by water infiltration. By approximately 3 ns, the CO_2_ domain shows signs of fragmentation, and water begins to penetrate from the mineral interface toward the confined CO_2_ channel. This invasion continues throughout the trajectory, and by 15 ns, a continuous aqueous bridge has formed, linking the water layers on the upper and lower kaolinite surfaces. The resulting configuration indicates that, although kaolinite is hydrophilic, the interfacial restructuring pathway differs substantially from that of hydroxylated silica, reflecting the influence of surface lattice geometry, hydrogen-bonding motifs, and mineral–fluid interaction strength.

Overall, the interfacial behaviors observed in the three mineral systems demonstrate that surface chemistry plays a decisive role in governing CO_2_–water organization under confinement. Hydrophilic hydroxylated silica promotes rapid spreading of water along the pore walls and results in pronounced phase separation, whereas the methyl-functionalized system drives the opposite outcome, stabilizing a CO_2_-wet configuration with a persistent encapsulated water droplet. Kaolinite, despite also being hydrophilic, follows a distinct restructuring pathway in which complete initial surface wetting is followed by gradual CO_2_ fragmentation and late-stage formation of a continuous aqueous network. These contrasting interfacial responses highlight that wettability alone is insufficient to predict fluid arrangement; instead, mineral lattice structure, hydrogen-bonding topology, and confinement geometry jointly determine the final equilibrium configuration. Such differences are critical for understanding storage security, capillary trapping mechanisms, and fluid migration during geological CO_2_ sequestration.

**Figure 3:**
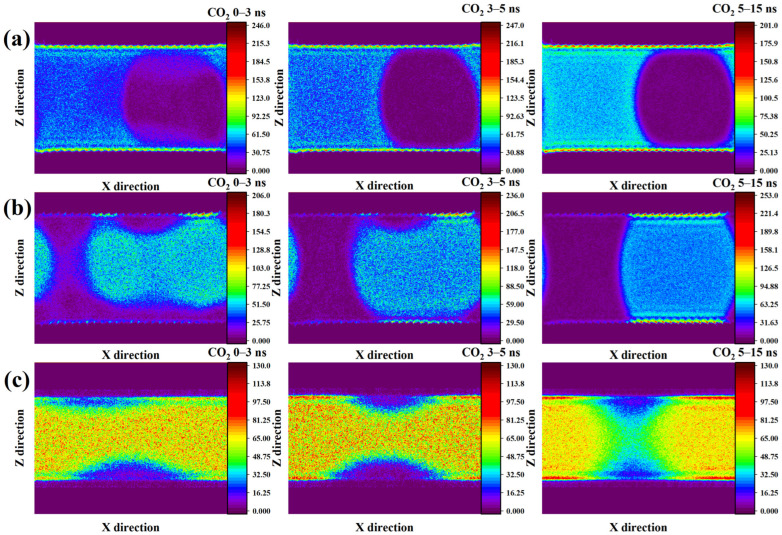
Temporal evolution of the two-dimensional density distribution of CO_2_ within (**a**) methylated and (**b**) hydroxylated SiO_2_ systems, and (**c**) within the kaolinite system.

To further quantify the spatial redistribution of CO_2_ observed in the structural snapshots, two-dimensional density maps were generated for each mineral system over the course of the simulation. Figure 3 shows two-dimensional number-density contour maps obtained by time-averaging molecular positions over selected simulation intervals. The color scale represents number density (nm^−3^), as indicated in each figure. The reported time windows (0–3 ns, 3–5 ns, and 5–15 ns) correspond to averaged density distributions over the entire interval, reflecting the temporal evolution of interfacial structure rather than instantaneous configurations. For the methylated SiO_2_ system (Figure 3a), the evolution of the CO_2_ density field reveals a clear surface-driven redistribution process. During the initial stage (0–3 ns), CO_2_ rapidly establishes a dense adsorption layer along the mineral surface, with the highest density concentrated near the pore walls and gradually decreasing toward the central region. A distinct low-density spherical zone appears on the right side of the pore, corresponding to the water cluster, where only sparsely distributed CO_2_ is present, though a few molecules remain dissolved within the interior of the droplet. As the system evolves from 3 to 5 ns, CO_2_ within the water domain becomes progressively depleted, while CO_2_ in the surrounding region becomes more uniformly distributed, sharpening the boundary between the droplet and the CO_2_-rich phase. In the later stage (5–15 ns), the density map becomes highly homogeneous outside the water droplet, reflecting a stable CO_2_-wet configuration and confirming that complete phase separation has been achieved.

In the hydroxylated SiO_2_ system (Figure 3b), the CO_2_ density distribution exhibits a markedly different evolution from that observed in the hydrophobic pore environment. At the beginning of the simulation, CO_2_ forms an irregular block-like cluster with a heterogeneous internal density, while only a weak and discontinuous adsorption layer develops along the mineral surface. Unlike the dense CO_2_ accumulation observed on the methylated surface, the interfacial density here remains comparatively low, although regions directly adjacent to the CO_2_ cluster show slightly higher adsorption intensity. Between 3 and 5 ns, the initially dispersed cluster contracts and gradually transforms into a more cylindrical structure. As its contact area with the hydroxylated surface increases, the near-wall CO_2_ density becomes more pronounced and expands laterally. During the later stage of the trajectory (5–15 ns), the system reaches a stable phase-separated configuration, with the left portion of the pore dominated by the aqueous phase and the right region fully occupied by CO_2_, confirming a wettability-governed rearrangement process.

For the kaolinite system (Figure 3c), the evolution of CO_2_ density follows a distinct pathway compared with both silica cases. At the initial stage, CO_2_ occupies most of the slit pore and is distributed relatively uniformly, with the exception of two cone-shaped low-density zones located adjacent to the kaolinite surfaces. These regions correspond to water-dominated domains, consistent with the early hydration behavior observed in the structural snapshots. Between approximately 3 and 5 ns, the CO_2_ phase becomes progressively denser, reflecting compaction and redistribution under confinement. A subtle increase in CO_2_ density is also detected near the mineral interface, suggesting partial adsorption driven by mineral–fluid affinity. During the later period (5–15 ns), the two low-density zones expand and eventually merge, dividing the CO_2_ region into two separate domains. This transition is consistent with the formation of a continuous water bridge spanning the pore. Notably, CO_2_ concentration decreases as the interface approaches the aqueous pathway, whereas the highest CO_2_ density remains near the kaolinite surfaces, albeit forming a relatively dilute interfacial layer. This behavior reflects a balance between hydrophilic mineral–water interactions and confinement-driven CO_2_ structuring.

**Figure 4:**
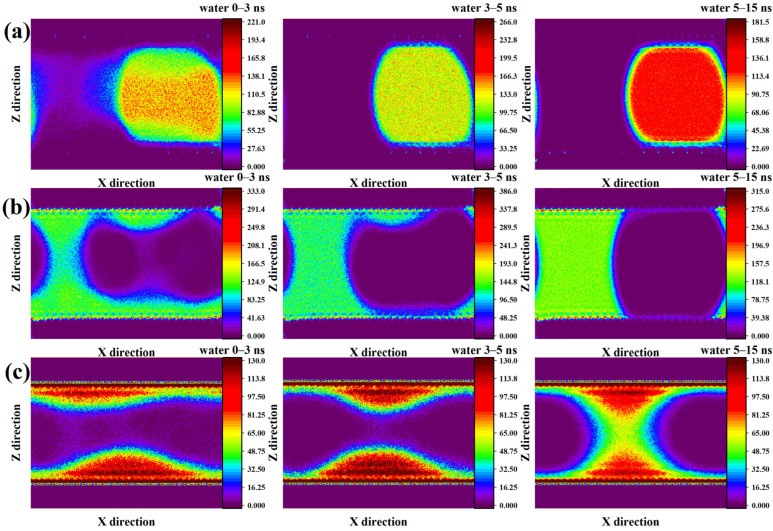
Temporal evolution of the two-dimensional density distribution of water within (**a**) methylated and (**b**) hydroxylated SiO_2_ systems, and (**c**) within the kaolinite system.

Figure 4 illustrates how the two-dimensional water density field varies with time in the three mineral systems. In the methylated SiO_2_ system (Figure 4a), the water molecules initially undergo gradual compression during the first 3 ns, forming an irregular spherical cluster with a non-uniform density distribution. The central region of the cluster exhibits a noticeably higher density, which decreases toward the gas–water interface. The left boundary of the cluster appears slightly elongated, suggesting uneven structural relaxation. Based on the density contours, little evidence of water adsorption on the SiO_2_ surface is observed at this stage. Between 3 and 5 ns, the water cluster becomes more compact, and its density distribution progressively homogenizes. The boundaries of the cluster also appear smoother, indicating enhanced structural stability. From 5 to 15 ns, the core density continues to increase significantly, while a sharp decline in density emerges at the gas–water interface. This trend implies further molecular packing within the bulk region and limited interaction with the hydrophobic surface.

In the hydroxylated SiO_2_ system (Figure 4b), water molecules show a strong tendency to adsorb onto the solid surface, forming a continuous hydration layer of measurable thickness. During the initial 0–3 ns, a water bridge develops between the upper and lower adsorption layers, connecting them across the pore space. As the simulation progresses, this bridge gradually expands, while the adsorption layer in the CO_2_-occupied region becomes progressively thinner and eventually disappears. During the final 5–15 ns segment, water near the CO_2_–SiO_2_ interface is fully displaced, resulting in a well-defined phase boundary. The left region becomes dominated by a densely packed and uniform water layer, whereas the right region contains almost no water molecules, indicating complete wetting asymmetry and a stabilized phase-separated configuration.

In the kaolinite system (Figure 4c), the strong hydrophilicity of the mineral surface leads to the rapid formation of a dense and highly concentrated water adsorption layer during the first 0–3 ns. A peak-shaped distribution of water emerges near the surface, with density gradually decreasing toward the pore center. Between 3 and 5 ns, these peak-like structures on the upper and lower surfaces become more pronounced and progressively converge. By 5–15 ns, the two water peaks fully connect, dividing the CO_2_ region into two separate domains. However, the connecting segment remains relatively dilute, exhibiting a density gradient that decreases from the kaolinite surface toward the gas–water interface, where the water density reaches its minimum.

Overall, the observed differences indicate that mineral surface chemistry governs water structuring and phase stability, where hydrophobic surfaces confine water into isolated droplets, moderately hydrophilic surfaces promote asymmetric wetting and interfacial rearrangement, and strongly hydrophilic clay surfaces facilitate continuous water film formation and bridge development that ultimately dissects the CO_2_ domain.

**Figure 5:**
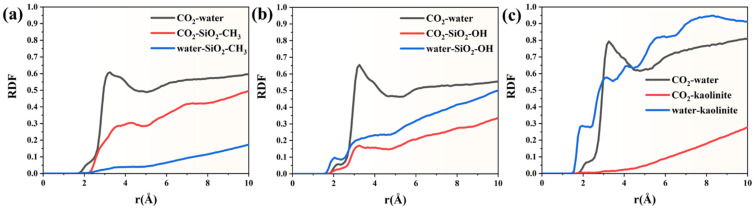
Radial distribution functions between different species within (**a**) the methylated SiO_2_ system, (**b**) the hydroxylated SiO_2_ system, and (**c**) the kaolinite system.

To further characterize molecular interactions at the fluid–solid interface, the radial distribution functions were calculated for key interacting species in the three mineral systems. The radial distribution trends reveal clear interfacial organization governed by hydrophobic mineral properties. The CO_2_–water RDF remains close to zero up to approximately 1.6 Å, indicating negligible short-range structural association and confirming poor mutual affinity at the interface. A detectable peak first appears around 1.75–2.15 Å, where the RDF increases from 0.0022 to 0.057, reflecting the onset of weak intermolecular arrangement. However, even beyond 3.0 Å, the RDF fluctuates around 0.55–0.60, showing no sharp maxima. This pattern suggests that CO_2_ and water remain largely phase-separated rather than forming structured hydration layers. In contrast, the RDF between CO_2_ and the methylated silica surface rises much more steeply. The first measurable values emerge at 2.05 Å (≈3.5 × 10^−5^), followed by a rapid increase to 0.169 at 2.85 Å and 0.239–0.285 between 3.15 and 3.75 Å. The absence of a narrow peak and the gradual slope imply diffuse adsorption rather than lattice-matched anchoring, typical for interactions dominated by van der Waals forces rather than hydrogen bonding. Beyond 4 Å, the CO_2_–surface RDF continues rising and reaches 0.452–0.50 near 8.95–10.05 Å, suggesting progressive layering parallel to the pore wall. Water interactions with the hydrophobic silica surface remain minimal throughout the full distance range. The RDF only begins to rise at ~1.55 Å (1.1 × 10^−4^) and remains substantially lower than that of CO_2_–surface coordination. Even at larger separations, values stabilize around 0.17–0.18 near 10 Å, nearly three-fold lower than the corresponding CO_2_–surface RDF. This disparity confirms that water is progressively excluded from the solid interface and does not form a persistent hydration layer (Figure 5a).

In the hydroxylated SiO_2_ system (Figure 5b), the three RDF profiles reveal clear structural differentiation among molecular interactions, reflecting the strong polarity and hydrogen-bonding nature of the surface. The CO_2_–water curve develops its first noticeable peak within 2.85–3.15 Å, reaching a height of approximately 0.64, indicating that CO_2_ molecules form a relatively loose but stable hydration environment. Although the coordination is not highly directional, the gradual decay toward a plateau suggests that CO_2_ behaves as a dispersed solute rather than forming localized clusters near the interface. In contrast, the CO_2_–surface interaction is much weaker. The first broad peak of the CO_2-_hydroxylated SiO_2_ RDF appears around 2.85–3.05 Å, with a significantly lower intensity (~0.15–0.17). This weak signal implies that CO_2_ experiences only minor van der Waals attraction and does not form persistent or ordered adsorption layers at the solid interface. Such behavior is consistent with the hydrophilic nature of the hydroxylated substrate, whose surface sites preferentially interact with polar species rather than nonpolar gases. The strongest ordering occurs in the water–hydroxylated SiO_2_ RDF. A sharp primary peak emerges between 1.65 and 1.85 Å, with values ranging from 0.07 to 0.15, indicating robust water–surface binding, most plausibly through hydrogen bonding. Notably, instead of rapidly decaying, this curve displays a progressive increase toward the bulk region, reaching values above 0.50 and rising gradually to ~0.56 at 10 Å. This trend demonstrates the formation of extended interfacial structuring rather than a single adsorption layer, suggesting multilayered hydration stabilized by cooperative hydrogen bonding.

The RDF profiles obtained for the kaolinite system reveal a distinctly ordered interfacial environment driven by the strong hydrophilicity and layered crystal structure of the mineral (Figure 5c). The water–kaolinite curve exhibits the earliest and most intense signal among the three interactions. A sharp rise appears at approximately 1.55–1.85 Å, reaching values above 0.22, and rapidly evolves into a pronounced peak of nearly 0.56 at 2.95 Å. This peak intensity exceeds that observed in the hydroxylated SiO_2_ case, highlighting the superior affinity of kaolinite toward water. Beyond the first coordination shell, the water–surface structure does not collapse; instead, it continues increasing steadily, surpassing 0.90 near 7 Å and eventually approaching ~0.91–0.95 in the bulk-like region (~9–10 Å). This progressive layering suggests the development of a dense, multi-shell hydration network stabilized by persistent hydrogen bonding and surface anchoring sites, consistent with kaolinite’s well-known ability to sequester water at its basal and edge surfaces. The CO_2_–water RDF follows a moderate organization pattern. A clear first maximum emerges near 3.05–3.25 Å, with a peak height close to 0.79, indicating that CO_2_ becomes surrounded by a relatively structured hydration shell. However, compared with water–kaolinite ordering, this signal is less intense and more diffuse, meaning CO_2_ remains largely solvated rather than adsorbing at the mineral surface. The gradual decay toward the long-range limit further implies that CO_2_ remains dynamically dispersed and does not participate in strong clustering or interfacial confinement. By contrast, CO_2_–kaolinite interactions are minimal. The first observable peak appears only around 2.75–3.35 Å, with maximum values below 0.02, which is almost an order of magnitude weaker than CO_2_–water coupling. This very small amplitude suggests negligible surface attraction: CO_2_ does not establish a stable adsorption layer nor form specific binding configurations with the mineral lattice. Instead, the hydration layers surrounding kaolinite effectively displace CO_2_ away from the interface, preventing direct mineral–solute contact.

**Figure 6:**
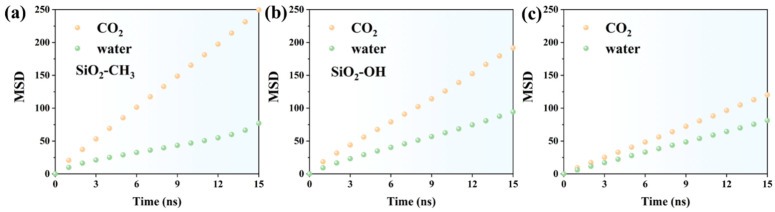
Mean square displacement (MSD) of CO_2_ and H_2_O molecules in (**a**) methylated SiO_2_, (**b**) hydroxylated SiO_2_, and (**c**) kaolinite systems.

To further elucidate the mobility of fluids at the mineral–fluid interface, the mean square displacement (MSD) profiles of CO_2_ and H_2_O were examined in three representative systems: methylated silica, hydroxylated silica, and kaolinite. The MSD values in the methylated SiO_2_ system reveal a clear mobility contrast between CO_2_ and water over the 15 ns simulation period (Figure 6a). At 1 ns, CO_2_ exhibits an MSD of 20.64, which is already more than twice that of water (9.95), suggesting that CO_2_ molecules experience weaker confinement at the hydrophobic surface. As time progresses, this mobility gap continues to widen. By 5 ns, CO_2_ reaches 85.38, while water only increases to 28.92, meaning CO_2_ diffuses at roughly three times the rate of water. The trend becomes even more pronounced in the latter simulation stage. At 10 ns, CO_2_ achieves an MSD of 165.27, compared to water at 46.88, indicating persistent molecular freedom and limited intermolecular resistance. By the end of the simulation at 15 ns, CO_2_ reaches 249.44, whereas water remains comparatively restricted at 77.00. This final ratio shows that CO_2_ movement is more than three-fold higher, reinforcing that the methylated surface fails to immobilize water or restrict CO_2_ penetration.

In the hydroxylated SiO_2_ system (Figure 6b), both CO_2_ and water exhibit continuous diffusion behavior throughout the 15 ns trajectory, but the contrast in their mobilities is narrower compared to the methylated surface. At 1 ns, CO_2_ reaches an MSD of 18.48, while water attains 9.27, giving an initial mobility ratio of approximately 2:1. This gap persists but does not diverge as sharply as observed in the hydrophobic system, implying stronger interfacial constraints for CO_2_ and enhanced water–surface affinity due to hydrogen bonding. As the simulation progresses, both species continue increasing in MSD with time. At 5 ns, CO_2_ records 67.59, and water reaches 34.86, maintaining a comparable diffusion ratio. By 10 ns, CO_2_ reaches 126.13, whereas water reaches 62.54, again demonstrating nearly proportional growth. This proportional increase is indicative of a surface environment where neither species experiences complete immobilization, but both interact with the hydroxyl groups to varying degrees. At the final simulation interval, 15 ns, CO_2_ rises to 191.59, while water reaches 94.35. The fact that CO_2_ mobility remains higher yet not excessively dominant suggests that the hydroxylated silica surface partially restricts CO_2_ diffusion through polar interactions, while simultaneously facilitating water structuring and motion via hydrogen bonding networks. Compared to the methylated system, the reduced CO_2_ diffusivity and enhanced water MSD values reflect a shift toward a more hydrophilic environment where interfacial forces exert measurable regulatory effects on molecular transport. The linear MSD evolution further supports sustained diffusive motion rather than adsorption-limited behavior.

In the kaolinite system (Figure 6c), both CO_2_ and water exhibit steady diffusion over time, yet the mobility contrast between the two phases narrows significantly compared with the silica-based environments. At 1 ns, the MSD values reach 9.41 for CO_2_ and 6.03 for water, indicating only a modest difference in transport behavior. This early-stage trend suggests that both species experience strong interaction forces with the kaolinite surface, likely dominated by hydroxyl-mediated hydrogen bonding and electrostatic attraction. As the simulation continues, diffusion increases in a near-linear fashion for both components. At 5 ns, CO_2_ reaches 40.71, while water records 27.76, maintaining a relatively constrained mobility ratio compared to the more hydrophobic surfaces. By 10 ns, CO_2_ reaches 80.56 and water 53.81, further demonstrating that neither species undergoes rapid escape from the interfacial region. Instead, their motion appears progressively governed by the highly hydrophilic nature of kaolinite, which stabilizes structured hydration layers. At the final simulation stage (15 ns), the MSD values increase to 120.19 for CO_2_ and 81.18 for water. Although CO_2_ retains greater overall displacement, both sets of values are comparatively lower than those observed in the silica systems, reinforcing the interpretation that kaolinite imposes the strongest diffusive confinement. The persistent gap between CO_2_ and water, without plateauing behavior, indicates stable—but not immobilizing—interfacial interactions.

Overall, the MSD results reveal distinct mobility regimes governed by surface chemistry. The methylated SiO_2_ system exhibits the weakest interfacial confinement, allowing CO_2_ to diffuse rapidly while water remains comparatively restricted, resulting in the largest mobility contrast among the three systems. In contrast, the hydroxylated SiO_2_ interface moderates this disparity; both CO_2_ and water maintain continuous diffusion but with strengthened surface coupling, leading to a more balanced transport response as hydrogen-bonding interactions become more influential. The kaolinite system presents the strongest confinement effect, where both species show substantially lower MSD values and a notably reduced mobility gap. This behavior indicates that the hydrophilic mineral framework, together with its dense surface hydroxyl environment, stabilizes interfacial water layers and suppresses CO_2_ motion through persistent molecular interactions. Taken together, these observations demonstrate that increasing mineral hydrophilicity systematically transitions transport behavior from free diffusion toward structured, interaction-controlled mobility, with kaolinite representing the most restrictive boundary for both water and CO_2_ at the mineral–fluid interface.

**Figure 7:**
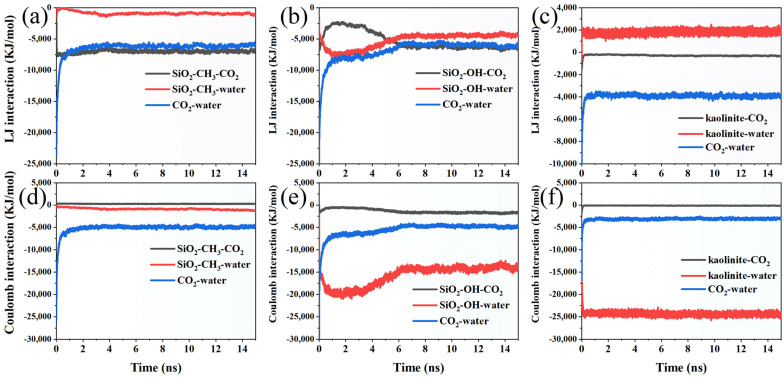
Lennard–Jones interaction energies among different molecular components within (**a**) the methylated SiO_2_ system, (**b**) the hydroxylated SiO_2_ system, and (**c**) the kaolinite system. (**d**) Coulombic interaction energies among the same components in (**e**) hydroxylated SiO_2_ and (**f**) kaolinite systems.

To gain deeper insight into the energetic drivers governing the interfacial behavior of fluids under supercritical CO_2_ conditions, the Lennard–Jones and Coulombic interaction energies among the different components were evaluated across the three representative mineral environments. The LJ energy between the methylated SiO_2_ surface and CO_2_ remains consistently strong and negative (~−7500 to −7000 kJ/mol), indicating a stable and persistent affinity of supercritical CO_2_ toward the hydrophobic interface (Figure 7a). Minor oscillations are observed, particularly in the early stage (<3 ns), which likely reflect the adjustment of CO_2_ density and layering before reaching a quasi-equilibrium state. After approximately 5 ns, the values plateau with reduced fluctuations, implying that the interfacial structuring of CO_2_ becomes relatively stable. In contrast, the LJ interaction between SiO_2_ and water is weaker (typically −300 to −1200 kJ/mol) and shows a clearer downward shift with time. The gradual increase in interaction magnitude (more negative) suggests progressive displacement of water molecules by CO_2_ at the hydrophobic surface, leading to an increasingly unfavorable water–solid contact environment. This behavior aligns with the expected wetting characteristics of a methylated surface, where water progressively retracts rather than forming a stable interfacial layer. The CO_2_–water LJ interaction exhibits the largest magnitude change (from ~−13,700 kJ/mol initially to ~−5800–6500 kJ/mol after stabilization), representing the strongest reduction among all pairs. The sharp decline during the first ~4 ns indicates a transition from a mixed fluid configuration toward a more phase-segregated state, where CO_2_ and water begin forming distinct domains. Beyond ~5 ns, the energy stabilizes at a lower level, suggesting a persistent interfacial arrangement with reduced cross-phase collisions and more demixed molecular organization.

The LJ interaction energies reveal a clear temporal evolution of molecular affinity at the hydroxylated SiO_2_–fluid interface. At the beginning of the simulation (≈0.1 ns), CO_2_ exhibits the strongest interaction among all pairs, with an energy of approximately −15,500 kJ/mol, reflecting strong confinement and dense packing near the surface (Figure 7b). However, this interaction progressively weakens over time and stabilizes around −6000 to −5500 kJ/mol after ~9 ns, indicating a gradual structural relaxation and reduced clustering of CO_2_ molecules. In contrast, the water–surface interaction becomes increasingly dominant. From an initial value near −4630 kJ/mol, it becomes more negative and plateaus close to −4300 kJ/mol between 10 and 15 ns, suggesting that water molecules rearrange to form a persistent hydration layer on the hydroxylated surface. A similar trend is observed for the water–CO_2_ interaction pair: the interaction strength decreases from approximately −13,000 kJ/mol at early stages to a narrower range between −6300 and −5800 kJ/mol in the final 5 ns. This reduction implies weakened direct CO_2_–water associations as interfacial structuring progresses and competitive adsorption favors water–surface affinity. Notably, after ~8 ns, all interaction curves exhibit reduced fluctuations, signaling the formation of a quasi-steady interfacial configuration. Overall, the quantitative patterns demonstrate a transition from a CO_2_-dominated early adsorption regime to a water-stabilized surface environment, driven by hydrogen bonding and surface wettability.

The Lennard–Jones interaction profiles demonstrate distinct interfacial affinity evolution among kaolinite, CO_2_, and water throughout the 0–15 ns simulation (Figure 7c). At the initial stage (≈0.1 ns), the CO_2_–water interaction dominates, reaching −5379 kJ/mol, indicating strong molecular aggregation driven by dispersion forces. This interaction gradually becomes less negative and fluctuates around −3800 to −4100 kJ/mol after 5 ns, suggesting partial dispersion of CO_2_ molecules and weakened clustering as the system approaches equilibrium. In contrast, the water–kaolinite interactions remain consistently positive (≈1300–2200 kJ/mol), reflecting a persistent repulsive tendency between interfacial water and the mineral surface. Notably, this value stabilizes near 1800–2100 kJ/mol after ~6 ns, implying the formation of a relatively stable hydration structure rather than dynamic reorganization. Meanwhile, the CO_2_–kaolinite interaction energy remains comparatively weak, varying between about −160 and −360 kJ/mol. Although a minor trend toward more negative values is observed with time, this behavior reflects only a modest increase in interfacial affinity, rather than pronounced CO_2_ adsorption onto the kaolinite surface. The combined trends indicate a competitive adsorption mechanism in which water establishes a stable interfacial layer first, while CO_2_ primarily interacts with water clusters and only weakly associates with the mineral surface. By the final simulation stage, all three interactions show reduced fluctuation amplitudes, marking the transition of the system from a dynamically adjusting state to a quasi-steady interfacial configuration.

The Coulombic interaction profiles reveal distinct interfacial electrostatic behavior at the CO_2_–water-methylated silica interface throughout the 0–15 ns simulation (Figure 7d). The interaction between CO_2_ and water shows the strongest magnitude, initially reaching −11,138 kJ/mol, reflecting a highly cohesive CO_2_–H_2_O network. This value rapidly becomes less negative within the first nanosecond, stabilizing around −4800 to −5500 kJ/mol after ~5 ns, indicating partial restructuring of the hydrogen-bond network as CO_2_ becomes more dispersed rather than forming dense clusters. In contrast, the electrostatic interaction between methylated SiO_2_ and water remains consistently negative, ranging from −300 to −1325 kJ/mol, and becomes increasingly stronger over time. The gradual decrease to more negative values suggests progressive water alignment and partial ordering near the weakly hydrophobic surface, rather than classical strong adsorption behavior. The interaction intermittently intensifies after 10 ns (down to ~−1200 kJ/mol), implying that interfacial water layering becomes more structured as the system approaches equilibrium. The SiO_2_–CO_2_ interaction remains comparatively weak and positive throughout the trajectory (≈200–350 kJ/mol), reflecting persistent electrostatic repulsion between CO_2_ and the methylated surface. No significant monotonic trend is observed, though short-lived fluctuations suggest transient, non-binding encounters rather than stable adsorption. Overall, the energy hierarchy remains stable where |E(CO_2_–H_2_O)| ≫ |E(SiO_2_–H_2_O)| > |E(SiO_2_–CO_2_)|, demonstrating that electrostatic forces favor CO_2_ remaining hydrogen-bond-associated with water rather than interacting with the hydrophobic silica surface.

The Coulombic interaction profiles demonstrate a clear hierarchy among the three interacting pairs at the hydroxylated SiO_2_–CO_2_–water interface (Figure 7e). Throughout the 0–15 ns trajectory, the hydroxylated surface–water interaction exhibits the most negative electrostatic energy, starting from −15,800 kJ/mol at 0.1 ns and progressively intensifying to values fluctuating around −14,000 to −19,500 kJ/mol after equilibration. This consistently large magnitude indicates a strong and persistent hydrogen-bond network between surface hydroxyl groups and interfacial water molecules, reflecting high surface affinity and strong wettability. In comparison, the electrostatic interaction between CO_2_ and water also remains strongly negative but weaker than the SiO_2_–water interaction. The initial value of −1340 kJ/mol rapidly increases toward less negative values during the first 1 ns, stabilizing around −4500 to −7000 kJ/mol after ~3 ns. The marked reduction (>60%) relative to the early stage suggests that CO_2_ progressively transitions from a strongly hydrogen-bond-regulated hydration shell toward more diffuse hydration configurations, consistent with gradual CO_2_ clustering or interface partitioning. Conversely, the hydroxylated SiO_2_–CO_2_ interaction remains the weakest among the three pairs. The electrostatic energy begins at −1400 kJ/mol, becomes less negative during the first nanosecond, and fluctuates within a narrow band of −500 to −2000 kJ/mol thereafter. The modest magnitude indicates that CO_2_ experiences only weak attraction mediated by surface hydroxyl groups, with no evidence of stable adsorption. Instead, the interaction suggests transient contact controlled by local solvation rather than direct chemisorption.

The kaolinite–water interaction remained the dominant electrostatic contribution, with values consistently ranging from −23,500 to −25,500 kJ/mol, and fluctuations remaining below 8%, indicating the formation of a highly stable hydration layer driven by strong hydrogen bonding and long-range electrostatic attraction (Figure 7f). In comparison, the CO_2_–water interaction energy remained significantly weaker, fluctuating within −2700 to −3500 kJ/mol, suggesting only transient and weak dipole–quadrupole interactions between dissolved CO_2_ molecules and surrounding water. The kaolinite–CO_2_ interaction showed the smallest magnitude (approximately −50 to −200 kJ/mol) and the highest relative variability (>50%), implying that CO_2_ adsorption on the mineral surface is dynamic, weakly bound, and easily disrupted during molecular rearrangement. Collectively, these results demonstrate a strong energetic preference for water–mineral binding over CO_2_ adsorption, which effectively limits CO_2_ access to reactive surface sites and governs the competitive interfacial interaction mechanism.

**Figure 8:**
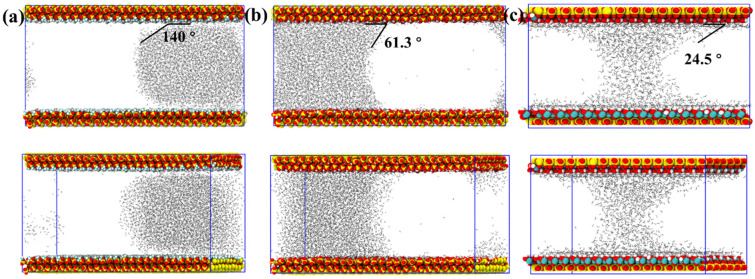
Interfacial morphologies and contact angles at 15 ns for (**a**) methylated SiO_2_, (**b**) hydroxylated SiO_2_, and (**c**) kaolinite systems.

To quantitatively characterize the wetting behavior at the mineral–water interface, we measured the contact angle of the confined water droplets by extracting the droplet boundary and fitting it with a circular arc using a MATLAB v. 2025-based algorithm. It should be noted that, due to the slit-pore geometry, the water phase is simultaneously in contact with both upper and lower mineral surfaces. Under such nanoconfined conditions, the classical macroscopic definition of contact angle is not strictly applicable. The reported angles, therefore, represent apparent geometric descriptors of interfacial equilibrium rather than true Young’s contact angles. The number of water molecules varies slightly among the models to ensure stable interfacial configurations and comparable filling degrees within pores of different surface chemistry, while the CO_2_/H_2_O ratio is maintained across all systems. For the methylated SiO_2_ surface (Figure 8a), the confined water cluster maintains a nearly spherical geometry and remains localized toward the right side of the slit. The MATLAB-fitted droplet boundary indicates a large contact angle of approximately 140°, and a distinct gap is observed between the water molecules and the solid surface. This incomplete adhesion reflects the weak water–surface affinity and is consistent with the dominance of hydrophobic interactions that suppress hydrogen-bond formation at the interface. In contrast, on the hydroxylated SiO_2_ surface (Figure 8b), water spreads more extensively and establishes tighter interfacial contact. The confined water adopts a cylinder-like morphology with a slight inward curvature in the central region, which arises from the increased hydrogen-bonding capacity of the hydrophilic SiO_2_ walls and the larger effective contact area with both upper and lower surfaces. The corresponding contact angle decreases markedly to 61.3°. Kaolinite exhibits an even stronger hydrophilic character, with a contact angle of only 24.5°. In this case, water forms a continuous bridge that links the upper and lower mineral surfaces, illustrating its pronounced tendency for interfacial wetting and structural coherence within the slit (Figure 8c).

In summary, the wettability contrast among the three mineral surfaces governs the geometric evolution of confined water and its interfacial adhesion behavior. Methylated SiO_2_, characterized by strong hydrophobicity, supports only loose water–surface contact and produces a highly non-wetting configuration with a contact angle near 140°. Hydroxylated SiO_2_ enhances hydrogen-bonding interactions, yielding tighter confinement, cylindrical droplet morphology, and a moderate contact angle of about 61.3°. Kaolinite exhibits the strongest hydrophilicity, as evidenced by a minimal contact angle of 24.5° and the formation of a continuous water bridge. These trends demonstrate that increasing surface polarity systematically promotes interfacial wetting, structural continuity, and the stability of water films within the slit.

## 4. Conclusions

This work provides an integrated molecular-scale picture of how supercritical CO_2_ and water behave at three representative mineral interfaces, e.g., methylated SiO_2_, hydroxylated SiO_2_, and kaolinite, under confined conditions relevant to subsurface sequestration. Across all systems, the interplay between surface chemistry, electrostatic interactions, and interfacial morphology determines the stability and mobility of the CO_2_–H_2_O mixture.

Quantitatively, the methylated SiO_2_ surface exhibits the weakest water affinity, reflected by a large water contact angle of ≈140° and a persistent nanoscale gap between the water cluster and the hydrophobic surface. The weak attraction is consistent with the relatively small magnitude of CO_2_–surface Coulomb interactions (typically within −400 to −1400 kJ/mol) and the modest CO_2_–H_2_O electrostatic coupling (around −6000 to −13,000 kJ/mol). As a result, water remains in a compact droplet, and CO_2_ preferentially populates the surrounding pore space. In contrast, the hydroxylated SiO_2_ interface shows markedly stronger hydration. Water spreads into a cylindrical configuration between the two walls, anchored by extensive hydrogen bonding, yielding a contact angle of ≈61.3°. This wetting enhancement correlates with the much stronger surface–water electrostatic attraction, consistently around −18,000 to −20,000 kJ/mol, while CO_2_–surface Coulomb interactions remain moderate. The stronger binding flattens the water profile and weakens the CO_2_ mobility in the central region.

Kaolinite displays the most hydrophilic behavior. The water film bridges the upper and lower surfaces, forming a continuous “water bridge,” with a contact angle reduced to ≈24.5°. The kaolinite–water electrostatic term is the largest among all systems, typically −23,000 to −25,000 kJ/mol, accompanied by moderately attractive CO_2_–water interactions (~−2800 to −3500 kJ/mol). These trends indicate that structural hydroxyl groups and the intrinsic layer charge of kaolinite create a highly polar environment that strongly stabilizes water and restricts CO_2_ intrusion.

Overall, the comparison reveals a consistent mechanistic sequence: hydrophobic SiO_2_ < hydrophilic SiO_2_ < kaolinite in terms of water affinity, interfacial cohesion, and electrostatic binding strength. These distinctions govern the morphology of confined fluids, their dynamic rearrangement, and the accessibility of reactive sites. By linking wetting geometry, interaction energies, and interfacial structure, this study clarifies how mineral surfaces modulate the stability of CO_2_–H_2_O mixtures at conditions relevant to geological storage, offering transferable insights for evaluating caprock sealing and mineral–fluid compatibility in subsurface carbon-storage environments.

From an engineering perspective, the molecular-scale insights obtained in this study have direct implications for geological CO_2_ storage. The strong dependence of interfacial stability on mineral wettability indicates that clay-rich and hydroxylated mineral assemblages are inherently more effective at sustaining water films and restricting CO_2_ migration. Such behavior favors enhanced caprock sealing and reduced leakage risk. These findings provide mechanistic support for reservoir screening, caprock evaluation, and potential surface-chemistry-based strategies aimed at improving long-term CO_2_ containment in subsurface storage systems.

## Data Availability

The original contributions presented in this study are included in the article. Further inquiries can be directed to the corresponding author.
